# Isolation and Genetic Enhancement of Nitrogen-Fixing Rhizobacteria for Promoting Growth in Maize

**DOI:** 10.3390/microorganisms14051067

**Published:** 2026-05-09

**Authors:** Wenjing Cui, Zhi Yang, Xuhui Meng, Xiaoyan Wang, Wenhao Chen

**Affiliations:** 1 Solid-State Fermentation Resource Utilization Key Laboratory of Sichuan Province, Faculty of Agriculture, Forestry and Food Engineering, Yibin University, Yibin 644000, China; w_j_cui@163.com; 2 Zhongnong Chuanda (Beijing) Environmental Protection Technology Co., Ltd., Beijing 100094, China

**Keywords:** rhizosphere, maize, nitrogen-fixing bacteria, stress tolerance, growth promotion, genetic modifications

## Abstract

Aiming to reduce synthetic fertilizer dependence and enhance soil fertility, this study isolated and characterized nitrogen-fixing bacteria from the maize rhizosphere. Nitrogen-free selective media were used for bacterial isolation, followed by detection of the *nifH* gene and nitrogenase activity. Phylogenetic identification was conducted via *16S rRNA* sequencing. Growth-promoting traits, stress tolerance, and pot-based plant inoculation effects were assessed. Genetic modification of strain GN8811 was performed to improve nitrogen fixation and growth promotion. Seven isolates that carried the *nifH* gene and exhibited nitrogenase activity were closely related to four genera. Several isolates showed phosphate solubilization, iron chelation, IAA production, or potassium solubilization, with GN2003 and GN8811 tolerating high salinity and variable pH. Inoculation with GN8811 promoted maize growth comparable to nitrogen fertilization, and its genetically modified derivative (Δ*nifL*:P*rpoD*) showed further improvement even under high nitrogen conditions. These findings highlight the potential of combining microbial screening with genetic engineering to develop efficient bioinoculants for sustainable maize cultivation.

## 1. Introduction

Global food demand is projected to rise sharply as the world population approaches 10 billion, driving intensive agricultural production that relies heavily on synthetic fertilizers to sustain high yields [1]. This input-intensive model consumes large amounts of fossil energy and finite mineral resources, while low nutrient-use efficiency leads to substantial losses of nitrogen (N) to the environment [2]. Excessive N fertilization accelerates soil acidification, salinization, and organic matter depletion, and it contributes to water eutrophication, greenhouse gas emissions, and biodiversity decline [1,2,3,4]. These ecological and soil degradation problems threaten long-term productivity and conflict with global goals for climate-smart and sustainable agriculture [5]. Biological nitrogen fixation (BNF) mediated by nitrogen-fixing bacteria offers a promising strategy to partially substitute synthetic N fertilizers, reduce energy consumption and N losses, and enhance soil health, thereby supporting the green transformation of agroecosystems [3,6,7].

Nitrogen-fixing bacteria used as biofertilizers encompass several major groups, including symbiotic rhizobia, associative (*Pseudomonas*) and endophytic (e.g., *Azospirillum*, *Herbaspirillum*, *Gluconacetobacter*, *Enterobacter*) diazotrophs, and free-living soil bacteria such as *Azotobacter* [8,9,10,11,12,13,14]. Numerous field and greenhouse studies have shown that inoculation with these nitrogen-fixing bacteria can increase N acquisition, stimulate root growth, and improve yields across diverse cropping systems, while allowing partial reduction in mineral N inputs [15,16,17]. In maize (*Zea mays* L.), which does not form classical nodules, increasing attention has been devoted to exploiting free-living, associative and endophytic N-fixing bacteria as biofertilizers [18]. Diazotrophs such as *Azospirillum* spp., *Herbaspirillum* spp., and plant-associated *Enterobacteriaceae* have been shown to colonize maize rhizosphere, root mucilage, and internal tissues, contributing fixed N and emitting phytohormones that enhance root architecture and nutrient uptake [17,19,20,21]. Field trials in different agroecological zones report that maize inoculated with selected N-fixing strains can achieve comparable or higher grain yield with over 30% reduction in N input, increase biomass and grain protein content [17,22,23]. Nevertheless, considerable variability among maize genotypes, soil types, and climatic regimes often leads to inconsistent responses [24,25,26]. The effectiveness of nitrogen-fixing bacterial inoculants is strongly modulated by soil factors such as drought, salinity, and pH, which subsequently limit crop growth, productivity, and quality [24,27]. In detail, salinity and osmotic stress can impair membrane integrity, enzyme function, and colonization ability, thereby decreasing BNF and plant benefits, especially in arid and coastal regions [28]. Soil pH strongly affects diazotroph survival and nutrient availability. Acidification due to excessive N fertilization often suppresses sensitive taxa and alters community composition [28,29]. Drought limits carbon flow from plants to the rhizosphere, decreases bacterial activity and reshapes bacterial community [30,31]. Critically, high levels of inorganic N in soil inhibit nitrogenase expression and activity through regulatory networks, leading to downregulation of *nif* genes and reduced BNF [32]. These interacting constraints explain why promising strains often perform inconsistently outside controlled conditions.

To enhance the stability and reliability of N-fixing biofertilizers, researchers are pursuing complementary strategies that integrate microbial ecology, strain selection, and genetic engineering. On the one hand, intensive screening programs and omics-guided approaches are being used to identify robust diazotrophic strains with enhanced tolerance to heat, salinity, acidity, and drought, as well as superior root colonization and plant-growth-promoting traits [33]. Consortia combining N-fixing bacteria with other plant growth-promoting rhizobacteria or mycorrhizal fungi are also being developed to increase functional redundancy and resilience under fluctuating field conditions [34,35,36]. On the other hand, molecular engineering of regulatory circuits controlling BNF aims to partially uncouple nitrogenase expression from classical ammonium- or nitrate-mediated repression [37,38]. In particular, targeted modification of the NifL–NifA regulatory system, including alterations in *nifL* to relieve its inhibitory control over *nifA* under high N, has been proposed and tested to sustain nitrogenase activity in the presence of elevated inorganic N while minimizing metabolic burden and maintaining redox balance [39,40]. These advances, together with improved formulation technologies and maize genotype–microbe matching, hold considerable promise for stabilizing the contribution of N-fixing bacteria to N supply and for enabling more predictable reductions in synthetic N fertilizer use in sustainable maize production systems [41,42].

The current study aims to systematically isolate and characterize beneficial nitrogen-fixing bacteria from the maize rhizosphere, focusing on their plant growth-promoting (PGP) capabilities, environmental resilience and stable nitrogen fixation capabilities even in the presence of higher environmental nitrogen concentrations. Our findings from this study not only identify promising indigenous nitrogen-fixing bacteria with multiple plant growth-promoting traits from the maize rhizosphere but also underscore the potential of targeted genetic modifications to optimize microbial inoculants for sustainable agriculture. This research highlights the importance of exploring microbial diversity in local agricultural ecosystems and harnessing biotechnological tools to maximize the benefits of these green helpers.

## 2. Materials and Methods

### 2.1. Soil Sample Collection

Maize rhizosphere soil samples were collected from the root zone (0–20 cm depth) of healthy maize plants in an agricultural field located in Gansu province (a severely arid region in China) during the flowering stage. Soil adhering tightly to the roots after gentle shaking was considered rhizosphere soil. The soil samples were placed in sterile plastic bags, stored on ice, and transported to the laboratory for immediate processing.

### 2.2. Isolation and Screening of Nitrogen-Fixing Bacteria

The isolation of nitrogen-fixing bacteria was performed using a three-round enrichment culture method on nitrogen-free media. Specifically, 10 g of rhizosphere soil was added to 90 mL of sterile Ashby’s nitrogen-free broth in a 250 mL conical flask. The culture was incubated at 30 °C with shaking at 180 rpm for 7 d. After the first round of incubation, 1 mL of the culture was transferred to fresh Ashby’s broth and incubated under the same conditions for another 7 d. This subculturing process was repeated for a third round. After the third enrichment, serial dilutions of the culture were prepared and spread onto Ashby’s nitrogen-free agar plates. The plates were incubated at 30 °C for 3–5 d. Colonies exhibiting distinct morphologies and mucoid characteristics, typical of nitrogen-fixing bacteria like *Azotobacter*, were selected and purified by repeated streaking on fresh Ashby’s agar plates. All isolates were stored in 20% glycerol at −80 °C for subsequent studies.

### 2.3. Molecular Identification and Phylogenetic Analysis

Genomic DNA from the seven isolates was extracted using a bacterial genomic DNA extraction kit. The *16S rRNA* gene was amplified via PCR using universal primers 27F (5′-AGAGTTTGATCMTGGCTCAG-3′) and 1492R (5′-GGTTACCTTGTTACGACTT-3′) [43]. A phylogenetic tree was constructed using the Maximum Likelihood method in MEGA X software (10.0.5) with bootstrap analysis based on 1000 replications [44]. All sequences were submitted to the NCBI GenBank database under submission number SUB16088807.

### 2.4. Detection of the nifH Gene and Assay of Nitrogenase Activity

The presence of the *nifH* gene, a key marker for nitrogen fixation, was detected by colony PCR amplification using specific primers Nh21F (5′-GCIWTYTAYGGNAARGG-3′) and Cy55Nh428R (5′-CCRCCRCANACMACGTC-3′) [45,46]. The acetylene reduction assay (ARA) was employed to measure nitrogenase activity [47]. Briefly, bacterial strains were cultured in nitrogen-free Ashby’s broth to the logarithmic growth phase. The cell density was adjusted to an OD_600_ of 0.5 as the pre-inoculum for further use. Then, 10 mL of each bacterial suspension was transferred to a 50 mL glass vial, sealed with a rubber stopper, and 10% of the headspace air was replaced with acetylene (C_2_H_2_). The vials were incubated at 30 °C for 2 h. Gas samples (200 µL) from the headspace were analyzed using a gas chromatograph equipped with a flame ionization detector to quantify the amount of ethylene (C_2_H_4_) produced. Nitrogenase activity was expressed as nmol C_2_H_4_ produced per mg of bacterial protein per hour.

### 2.5. In Vitro Evaluation of Plant Growth-Promoting (PGP) Traits

The plant growth-promoting traits of the bacterial isolates were qualitatively evaluated using standard plate-based assays. All the test strains were cultured in LB medium for 2 d and the value of OD_600_ of all tested strains was adjusted to 0.05. Indole production was assessed by spot inoculating bacterial strains (10 µL) on Ashby’s agar supplemented with 5 mM L-tryptophan and incubating at 30 °C for 72 h. The appearance of a pink-to-red halo after treatment with Salkowski’s reagent was considered indicative of indoles (including IAA) production [48]. To quantify IAA production, a quantitative assay was performed using an HPLC (LC-20Ai, Shimadzu Corporation, Tokyo, Japan) method. IAA standard (10 mg) was dissolved in methanol to 10 mL (1 g/L stock). The stock was diluted with mobile phase to 0, 2.5, 5, and 10 μg/mL working solutions. Chromatographic conditions: Mobile phase: methanol: 0.01% acetic acid (45:55, *v*/*v*), filtered through 0.45 μm. Flow rate: 1 mL/min; detection: 254 nm; column temperature: 30 °C; injection: 10 μL. Each working solution was analyzed, and the curve was constructed by plotting peak area vs. concentration (μg/mL). The regression equation and correlation coefficient were calculated. Culture broth (10 mL) was mixed with 10 mL water, then extracted three times with 20 mL ethyl acetate each. The combined organic phases were evaporated to near dryness at 45 °C, redissolved in the mobile phase to 10 mL, and filtered (0.45 μm). The filtrate was analyzed by HPLC, and IAA concentrations were calculated from the calibration curve [49]. Phosphate-solubilizing ability was determined on Pikovskaya’s agar containing insoluble tricalcium phosphate. 10 µL of culture liquid was added to the plate. Plates were incubated at 30 °C for 5 d, and the formation of a clear halo around the colonies indicated phosphate solubilization [50]. Siderophore production was evaluated using Chrome Azurol S (CAS) agar. Isolates were incubated at 30 °C for 3–5 d, and the development of a clear halo surrounding the colonies indicated siderophore secretion [51]. Potassium solubilization was examined by spot inoculation on a plate containing potassium-bearing minerals; 10 µL of culture liquid was added to the plate, followed by incubation at 30 °C for 7 d. The presence of a clear halo zone around colonies was considered positive for potassium solubilization [52].

### 2.6. Evaluation of Environmental Stress Tolerance

The tolerance of the bacterial isolates to abiotic stresses was evaluated by monitoring their growth under different salinity and pH conditions. The tested strains were cultured in LB medium at 37 °C with shaking at 180 rpm for 2 d. The bacterial cultures were then harvested by centrifugation and washed with 0.8% NaCl. The cell density was adjusted to an OD_600_ of 1 as the pre-inoculum for further use. Bacterial strains (5%, *v*/*v*) were inoculated into Ashby’s liquid medium supplemented with different concentrations of NaCl (1%, 5%, and 10% *w*/*v*). The cultures were incubated at 30 °C with shaking at 180 rpm. Similarly, Ashby’s liquid medium was adjusted to different pH levels (5, 8, and 10) using sterile HCl or NaOH solutions. Bacterial strains were inoculated and incubated as described above. Bacterial growth was monitored by measuring the optical density at 600 nm (OD_600_) at 24 h intervals for 3 d.

### 2.7. Genetic Modification and Transcriptional Determination

Strains, plasmids, and primers used in this work are listed in Table S1 in the supplemental material. The genetic modification procedure was performed as described previously [53]. The pJQ200SK was digested by a SmaI. The upstream and downstream homologous arms (600–800 bp) of the *nifL* gene were amplified by PCR and sequentially inserted into the sucrose-sensitive suicide plasmid pJQ200SK-Km using the In-Fusion cloning method, constructing the knockout vector pJQ200SK-Δ*nifL*. This vector was introduced into GN8811 via triparental conjugation (Figure S1). The recipient strain GN8811, the donor strain harboring the suicide plasmid pJQ200SK-Δ*nifL*, and the helper strain containing the helper plasmid pRK2013 were all cultured in LB medium to the mid-logarithmic phase, with an OD of approximately 0.6. A total of 500 µL of each bacterial culture was collected and centrifuged at 6000 rpm to remove the supernatant. The cell pellets were resuspended in physiological saline. The three strains were mixed at an equal ratio (1:1:1) and vortexed. After removing the supernatant, the bacterial lawn was transferred onto LB solid medium containing kanamycin. Once the bacterial lawn was air-dried, the plates were incubated upside down for two days to allow for gene exchange. The bacterial lawn was then subjected to gradient dilution, and the 10^−3^ and 10^−4^ dilutions were spread onto LB agar plates containing kanamycin and incubated. After 2 d, the colonies that grew were considered putative single-crossover mutants. These putative single-crossover mutants were subsequently streaked onto LB agar plates containing kanamycin and LB agar plates containing sucrose. Colonies that grew on LB kanamycin plates but failed to grow on LB sucrose plates were identified as positive single-crossover mutants. The positive single-crossover mutants were transferred to LB liquid medium containing sucrose and kanamycin and cultured for 2 d. After incubation, the cultures were subjected to gradient dilution, and the 10^−3^ and 10^−4^ dilutions were spread onto LB agar plates containing sucrose and kanamycin. Following 2 d of incubation, the resulting colonies were identified as double-crossover mutants. Subsequently, the *nifL* deletion mutant (Δ*nifL*) clones were further verified by PCR amplification using primer *nifL*-NF, *nifL*-NR, *nifL*-WF and *nifL*-WR. Furthermore, to constitutively express the *nifA* gene (a positive regulator), a DNA fragment containing the constitutive *rpoD* promoter was synthesized. This fragment, together with the upstream and downstream homologous arms of *nifL*, was inserted into SmaI-digested pJQ200SK to generate pJQ200SK-Δ*nifL*::P*rpoD*. This plasmid was used to replace the native promoter of *nifA* in the Δ*nifL* mutant background via a similar homologous recombination strategy described above, generating the engineered strain Δ*nifL*::P*rpoD*.

The transcript levels of *nifA* in the wild-type and mutant strains under low-nitrogen (0 mM NH_4_NO_3_) and high-nitrogen (10 mM NH_4_NO_3_) conditions were analyzed by semi-quantitative RT-PCR. The strains were cultured in Ashby’s nitrogen-free medium until the stationary phase (2 d). The cultures were then centrifuged at 12,000 rpm to remove the supernatant, and RNA was extracted using an RNA extraction kit (TaKaRa MiniBEST Universal RNA Extraction Kit, TaKaRa, Tokyo, Japan). Briefly, cells are homogenized or ground in liquid nitrogen to release nucleic acids into the lysis buffer. The lysate is then sequentially passed through a gDNA Eraser Spin Column to remove genomic DNA, followed by an RNA Spin Column to bind RNA, thereby achieving high-quality RNA extraction. The reverse transcription reaction was prepared by mixing 6 µL of Hifair^®^ AdvanceFast SuperMix (Yeasen, Shanghai, China) with total RNA (0.5 µg), and the final volume was adjusted to 20 µL with RNase-free water. The reverse transcription reaction was carried out at 55 °C for 5 min, followed by 85 °C for 5 s. Semi-quantitative RT-PCR amplification was performed under the following conditions: initial denaturation at 95 °C for 3 min; followed by 15 cycles of 95 °C for 30 s, 55 °C for 30 s, and 72 °C for 30 min; with a final extension at 72 °C for 2 min. Finally, the PCR amplification products were analyzed by gel electrophoresis, and the brightness of the target band was used to represent the relative transcription level.

### 2.8. Pot Experiment to Evaluate Plant Growth Promotion

The tested strains were cultured in LB medium at 37 °C with shaking at 180 rpm for 2 d. The bacterial cultures were then harvested by centrifugation and washed with physiological saline. The cell density was adjusted to an OD600 of 0.1 as the pre-inoculum for further use. Maize seeds (maize varieties Zhengdan 958, ZD958, Institute of Food Crops, Henan Academy of Agricultural Sciences) were surface-sterilized with 75% ethanol for 3 min and 2% sodium hypochlorite for 5 min, followed by rinsing thoroughly with sterile distilled water. The seeds were then coated with bacterial suspensions (OD_600_ ≈ 1.0) of the wild-type or engineered strains for 2 h. Seeds coated with physiological saline served as the control. The treated seeds were sown in pots (one plant per pot) filled with a sterile mixture of sand and vermiculite (2:1, *v*/*v*). The plants were grown in a greenhouse under a 16/8 h light/dark cycle at 25–28 °C. The following fertilizer treatments were applied: (1) 100% nitrogen (full N, 200 kg N/ha equivalent), (2) 80% nitrogen, (3) 0% nitrogen, and (4) 0% nitrogen + bacterial inoculation. Full P (80 kg/ha) and full K (70 kg/ha) were applied in all pot experiments. Each treatment consisted of at least three replicates, with no fewer than 10 plants per replicate. Plants were watered as needed with a nitrogen-free Hoagland nutrient solution, and irrigation was performed every 5 d throughout the experimental period. After 45 d of growth, plant height, root length, and the fresh weight of shoots and roots were measured to assess the plant growth promotion effects of the bacterial inoculations.

### 2.9. Bacterial Growth Curve Determination

Bacterial strains were cultured in LB and Ashby’s media for 2 d and then inoculated at 5% (*v*/*v*) into LB (nitrogen-rich) and Ashby’s (nitrogen-free) liquid media, respectively. The cultures were incubated at 30 °C with shaking at 180 rpm. Bacterial growth was monitored by measuring the optical density at 600 nm (OD_600_) at 3 h intervals for 2 d.

### 2.10. Data Visualization and Statistical Analysis

Data visualization was performed using GraphPad Prism 10 software. Statistical analysis of phenotypic data from bacterial strain inoculation, based on three independent biological replicates, was conducted using one-way ANOVA followed by Tukey’s test (α = 0.05) in SPSS 25. Significant differences are indicated by lowercase letters.

## 3. Results

### 3.1. Characterization and Nitrogen-Fixing Activity Assessment of Nitrogen-Fixing Bacteria from Maize Rhizosphere

**Figure 1:**
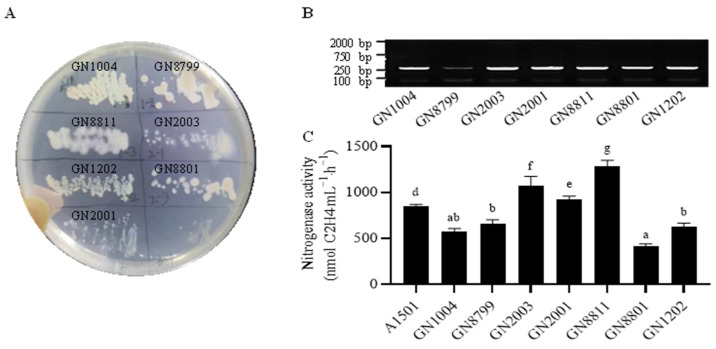
Characterization and nitrogenase activity assessment of isolated nitrogen-fixing strains. (**A**) The colony morphology of the isolated strains on Ashby’s medium; (**B**) The *nifH* amplification of isolated strains; (**C**) Nitrogenase activity determination of isolated strains. A1501, a well-studied nitrogen-fixing bacterium, was used as a positive control. Error bars represent SD. Significant differences between means were indicated by different letters based on ANOVA followed by Tukey’s test (α = 0.05).

Using a multi-step enrichment process on nitrogen-free medium, we isolated a total of seven bacterial strains from maize rhizosphere soil that were capable of stable growth under nitrogen-free conditions (Figure 1). All strains formed viscous bacterial lawns on the nitrogen-free medium and were designated as GN1004, GN8799, GN2003, GN8811, GN2001, GN8801, and GN1202 (Figure 1A). Further detection of the *nifH* gene, a key marker for nitrogen fixation, confirmed that all seven isolates possessed the *nifH* gene (Supplementary Table S1), with a core conserved fragment length of approximately 250 bp (Figure 1B). Moreover, nitrogenase activity was highest in GN8811, followed by GN2003, GN2001, GN8799, and GN1202 (which showed similar activities), and then GN1004 and GN8801 (which showed the lowest and similar activities) (Figure 1C). Additionally, A1501 (*Pseudomonas stutzeri*), a well-characterized nitrogen-fixing model strain, exhibited moderate nitrogenase activity (Figure 1C).

### 3.2. Phylogenetic Analysis of Isolated Nitrogen-Fixing Bacteria

**Figure 2:**
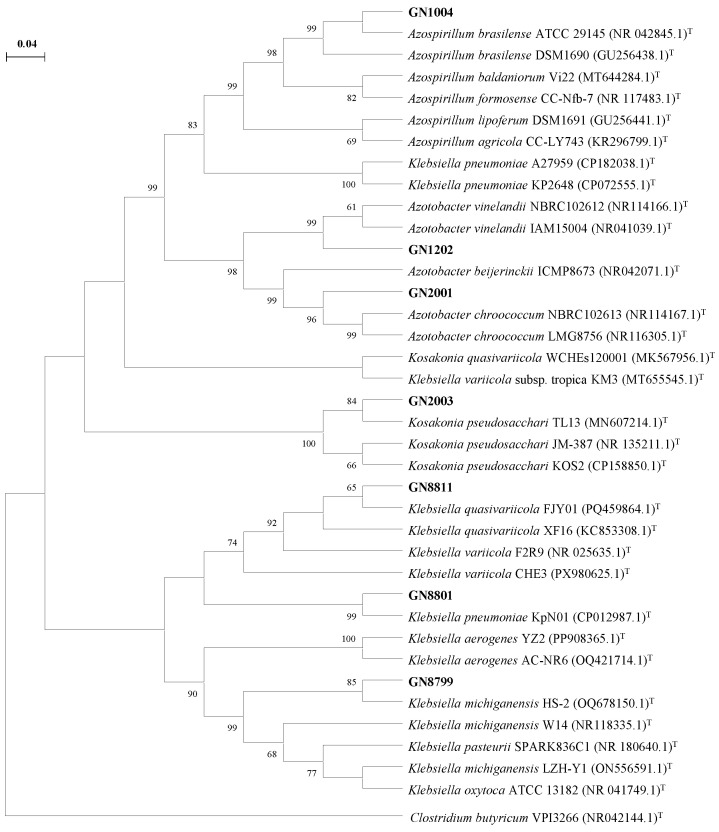
The phylogenetic tree based on *16S rRNA* genes of isolated strains. Maximum likelihood phylogenetic trees of 34 (7 candidates, 27 reference strains) *16S rRNA* sequences were constructed by Mega X software. Bootstrap values above 60% were shown at the branch points. Bar, 0.04 substitutions per nucleotide position. Accession numbers of the reference sequences from GenBank were given in brackets. T, type strains.

To elucidate the taxonomic status of the seven isolated strains, a phylogenetic analysis based on *16S rRNA* gene sequences was conducted. The results revealed that the seven strains were distributed across four genera, including *Azotobacter*, *Azospirillum*, *Kosakonia*, and *Klebsiella* (Figure 2), demonstrating a high level of microbial diversity. Specifically, strain GN2001 was closely related to *Azotobacter chroococcum*, GN1202 to *Azotobacter vinelandii*, GN1004 to *Azospirillum brasilense*, GN2003 to *Kosakonia pseudosacchari*, and GN8799 to *Klebsiella michiganensis*. GN8801 and GN8811 were closely related to *Klebsiella pneumoniae* and *Klebsiella quasivariicola* (Figure 2).

### 3.3. Evaluation of Plant Growth-Promoting Traits of the Isolated Strains

**Figure 3:**
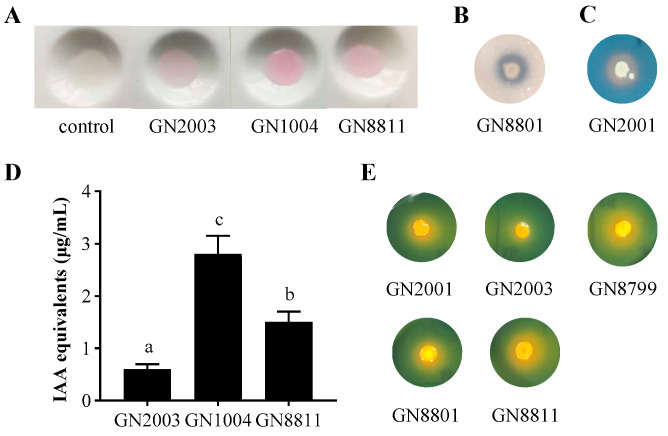
Qualitative assessment of plant growth-promoting characteristics of isolated strains under in vitro conditions. Assessment of qualitative (**A**) and quantitative (**D**) IAA production. Error bars represent SD. Significant differences between means are indicated by different letters based on ANOVA followed by Tukey’s test (α = 0.05). P solubilization (**B**), siderophore production (**C**) and K Solubilization (**E**) were qualitatively tested, respectively.

To comprehensively evaluate the plant growth-promoting potential of the seven isolated strains, their abilities to produce indoles (including IAA), solubilize phosphate, produce siderophores, and solubilize potassium were qualitatively assessed. The results indicated that strains GN1004, GN2003, and GN8811 exhibited indole production, as evidenced by the pink coloration on the white porcelain plate assay (Figure 3A). The quantitative assessment confirmed the IAA production levels of GN1004, GN8811, and GN2003 were 0.6, 1.5 and 2.7 µg/mL, respectively (Figure 3D). In terms of phosphate solubilization, only GN8801 formed a visible dissolution zone on the agar plate (Figure 3B). For siderophore production, solely GN2001 demonstrated clear iron-chelating ability, forming a distinct halo on the assay plate (Figure 3C). Regarding potassium solubilization, five strains—GN2001, GN8799, GN8801, GN8811, and GN2003—exhibited varying abilities to solubilize potassium, with GN2003 showing the weakest activity, indicated by a faint dissolution zone (Figure 3E).

### 3.4. Physiological Characterization of Acid, Alkali, and Salt Tolerance in the Test Isolates

**Figure 4:**
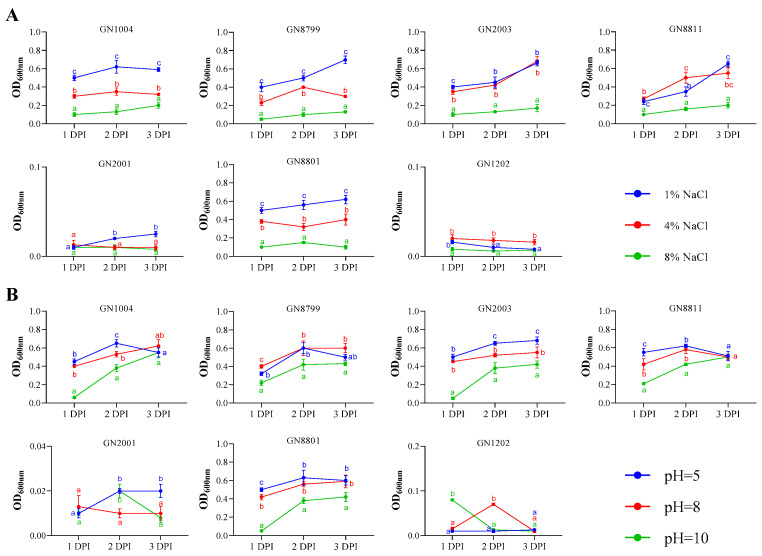
Effect of varying NaCl concentrations (**A**) and pH levels (**B**) on the growth of the tested strains. DPI, days post inoculation. Error bars represent SD. Significant differences between means are indicated by different letters based on ANOVA followed by Tukey’s test (α = 0.05). At least three replications of the experiment were carried out.

Furthermore, to assess the tolerance of the tested strains to adverse environmental conditions, their growth status was evaluated under different salt concentrations and pH levels. The results demonstrated that higher salinity significantly inhibited bacterial growth (Figure 4A). Specifically, at 10% NaCl concentration, biomass was markedly suppressed and showed a negligible increase over time. In contrast, at lower salt levels (1% and 5%), the biomass of all strains gradually increased with prolonged incubation, with greater biomass accumulation observed at lower salinity (Figure 4A). Notably, strains GN2001 and GN1202 exhibited the lowest salt tolerance, while GN2003 and GN8811 showed the highest. The remaining strains displayed intermediate tolerance without significant differences. Similarly, alkaline conditions (pH = 10) strongly inhibited the growth of most strains, resulting in a significant reduction in biomass (Figure 4B). In comparison, at pH 5 and pH 8, bacterial biomass increased substantially (Figure 4B). Among the tested strains, GN1004, GN8799, GN2003, GN8811, and GN8801 demonstrated better adaptation to varying pH conditions, whereas GN2001 and GN1202 were the most sensitive to acidic and alkaline stress (Figure 4B). Additionally, all strains exhibited better growth in LB (nitrogen-rich) medium than in Ashby’s (nitrogen-free) medium, as indicated by higher OD600 values (Figure S2). Meanwhile, compared with other strains, GN2001 and GN1202 showed poor growth in both LB (nitrogen-rich) and Ashby’s (nitrogen-limited) media, with even poorer growth observed in Ashby’s medium (Figure S2).

### 3.5. Inoculation Effects of Nitrogen-Fixing Bacterial Strains on Maize Growth

**Figure 5:**
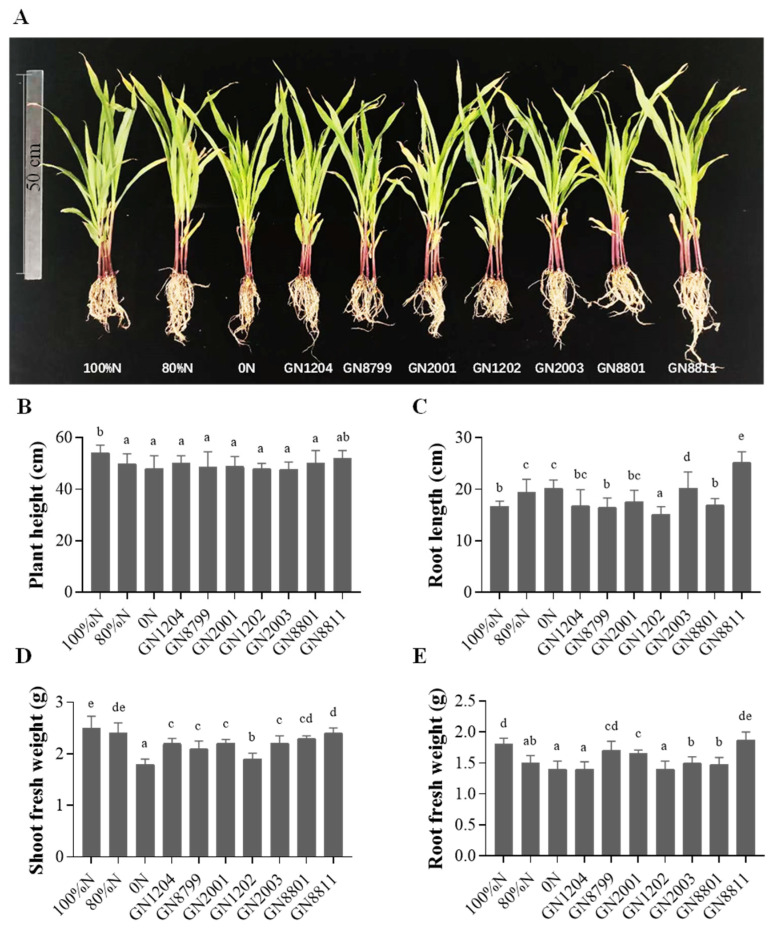
The inoculation effect of the tested strains on maize. (**A**) Growth and morphological phenotypes of maize plants treated with isolated strains. Shoot height (**B**), root length (**C**), shoot fresh weight (**D**) and root fresh weight (**E**) were measured to evaluate the effect of the inoculation performance of individual isolates on maize. Error bars represent SD. Significant differences between means are indicated by different letters based on ANOVA followed by Tukey’s test (α = 0.05). At least two additional replications of the experiment were carried out.

The plant growth-promoting effects of the nitrogen-fixing bacteria were evaluated through inoculation experiments. Higher nitrogen application levels generally resulted in improved plant growth, as reflected by increased plant height, as well as shoot fresh weights and root fresh weight (Figure 5). Overall, the growth performance of maize inoculated with strain GN8811 was comparable to that of the 100% nitrogen treatment, and even exceeded it in terms of root length and root weight (Figure 5C,E). Regarding plant height, all inoculation treatments performed similarly to the 80% N and 0% N treatments, while GN8811 inoculation achieved results equivalent to the 100% N treatment (Figure 5B). In terms of root length, both GN2003 and GN8811 significantly outperformed all nitrogen treatment groups and other inoculated groups (Figure 5C). For root weight, GN8799 and GN8811 reached levels comparable to the 100% N treatment, and GN8799, GN8811, and GN2001 all exceeded the 80% N treatment level, whereas the remaining inoculation treatments showed no significant difference from the 80% N treatment (Figure 5E). Regarding shoot fresh weight, all bacterial inoculations significantly outperformed the 0% N treatment. Among them, GN8801 and GN8811 reached the level of the 80% N treatment, though slightly lower than that of the 100% N treatment (Figure 5D).

### 3.6. Genetic Modification of GN8811 Enhanced Plant-Growth Promotion

**Figure 6:**
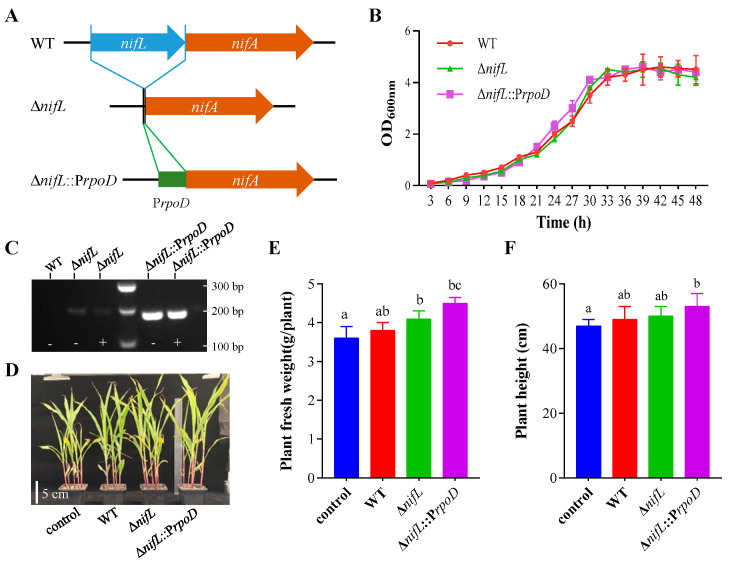
The GN8811 genetic modification enhances nitrogenase activity and plant growth. (**A**) Genetic modification model. WT, wild type; *ΔnifL*, *nifL* mutant; *ΔnifL*::P*rpoD*, *nifL* mutant compensates with *rpoD* promoter. (**B**), Growth curve of GN8811 and its derivatives under nitrogen-rich medium (LB). (**C**), The *nifA* transcript activity was detected through semi-quantitative PCR. The symbols − and + represent nitrate ammonium concentrations of 0 mM and 10 mM, respectively. Inoculation performance (**D**), plant fresh weight (**E**) and plant height (**F**) were used to evaluate the effect of the individual isolates on maize growth under nitrogen-free conditions. Error bars represent SD. Significant differences between means are indicated by different letters based on ANOVA followed by Tukey’s test (α = 0.05). At least two additional replications of the experiment were carried out.

To enhance the nitrogen-fixing capability of the outstanding strain GN8811, genetic modifications were performed to eliminate nitrogen fixation repression and strengthen *nifA* expression (Figure 6A). The wild-type (WT), Δ*nifL* mutant, and Δ*nifL*::P*rpoD* engineered strain showed comparable growth rates in nitrogen-rich and nitrogen-free culture medium (Figure 6B and Figure S3). Under high-nitrogen conditions, *nifA* expression was undetectable in the WT, while the Δ*nifL* mutant exhibited weak *nifA* expression under both low- and high-nitrogen conditions. In contrast, the Δ*nifL*::P*rpoD* strain demonstrated strong *nifA* expression regardless of nitrogen availability (Figure 6C). Further pot experiment results confirmed that inoculation with wild type GN8811 significantly promoted maize growth (Figure 6D), reflected by notable increases in fresh weight and plant height (Figure 6E,F). Moreover, inoculation with Δ*nifL*::P*rpoD* resulted in the highest plant fresh weight and height, although the difference from Δ*nifL* was not statistically significant in some comparisons (Figure 6E,F). Additionally, we inoculated maize with the GN8811 wild-type strain and its mutant alongside A1501 under nitrogen-free conditions. The results showed that although the GN8811 mutant (Δ*nifL*::P*rpoD*) exhibited no difference in plant height compared to A1501, its shoot fresh weight was significantly increased, suggesting a stronger nitrogen-fixing capacity (Figure S4).

## 4. Discussion

This study isolated seven indigenous nitrogen-fixing rhizobacteria from the maize rhizosphere, revealed considerable taxonomic and functional diversity among them, and identified GN8811 as an outstanding strain with strong nitrogenase activity, multiple plant growth-promoting traits, and broad environmental tolerance. Furthermore, targeted genetic engineering of GN8811 by deleting *nifL* and enhancing *nifA* expression markedly improved nitrogen fixation and maize growth promotion, even under high-N conditions. Together, these results demonstrate a dual strategy—ecological screening plus regulatory-circuit engineering—for developing robust microbial inoculants that can support maize production with reduced dependence on synthetic N fertilizers.

Our findings are broadly consistent with previous reports showing that maize rhizospheres harbor diverse diazotrophs, including *Azotobacter*, *Azospirillum*, *Klebsiella* and *Kosakonia*, which contribute to N supply and plant growth promotion through BNF and additional PGP traits such as phytohormone production and nutrient solubilization [54,55,56,57]. The identification of *Azotobacter*, *Azotobacter* and *Azospirillum* among our isolates agrees with studies showing these genera as common maize-associated diazotrophs with IAA production and nutrient-solubilizing capacities [58,59]. Our discovery of *Klebsiella*, together with *Kosakonia*, corroborates recent work highlighting *Enterobacteriaceae* as important plant-associated diazotrophs capable of colonizing maize roots and mucilage and promoting grain yield under reduced N fertilization [60,61]. Compared with previous studies, our work extends the characterization of maize-associated diazotrophs in several ways. First, we systematically evaluated multiple PGP traits across all isolates, showing clear functional differentiation: GN1004 had the strongest IAA production; GN8801 was the only strain with detectable phosphate solubilization; GN2001 uniquely produced siderophores, and several strains (including GN8801 and GN8811) solubilized potassium. Similar trait partitioning has been described in other PGPR consortia where different strains contribute complementary functions [62,63], suggesting that assembling GN2001, GN8801, GN8811 and others into tailored consortia may further enhance maize performance. Second, our evaluation of salt and pH tolerance revealed that GN2003 and GN8811 maintained relatively robust growth under 10% NaCl and across pH 5–10, which is comparable to or higher than tolerance levels reported for many diazotrophs used in saline or acidic soils [64,65]. Such broad tolerance is particularly relevant for regions facing soil salinization and acidification due to intensive N fertilization [66,67].

The inoculation experiments showed that GN8811, and to a lesser extent GN2003 and GN8799, substantially improved maize biomass, root length and root weight, achieving performance similar to or exceeding 100% N-fertilized controls for specific traits. These results align with previous field and pot studies where inoculation with *Azospirillum*, *Herbaspirillum* and *Enterobacteriaceae* enabled 20–30% reductions in mineral N without yield penalties [68,69], indicating a realistic potential for partial replacement (20–30%) of synthetic N inputs under controlled conditions. However, we also observed that not all isolates with the *nifH* gene and nitrogenase activity produced equally strong growth responses, and that different strains preferentially enhanced specific components (e.g., root length versus shoot biomass). Furthermore, other plant growth promotion traits of the strains showed no direct correlation with their performance in maize inoculation. This variability echoes earlier reports of inconsistent field responses driven by strain × genotype × environment interactions [70,71] and underscores the importance of matching inoculant strains to target environments and crop varieties. We also verified that the genetic modification of GN8811 enhanced the plant inoculation performance. Our work was consistent with previous studies that have proposed and demonstrated that altering *nifL* or engineering *nifA* expression can partially uncouple nitrogenase from classic ammonium/nitrate repression in diazotrophs [39,40,72], limiting the contribution of inoculants in fertilized fields. Our results also confirmed that a Δ*nifL*::P*rpoD* construct in a maize-associated *Klebsiella* strain maintains strong *nifA* expression and enhances plant growth under high nitrogen concentration. By showing that engineered GN8811 can still promote maize growth when mineral nitrogen is abundant, this indicates that rational modification of regulatory circuits can improve the reliability of N-fixing inoculants under realistic management regimes.

Despite these strengths, several limitations must be acknowledged. Our inoculation experiments were conducted under controlled pot conditions; although they simulate different N levels, they do not fully capture the complexity and variability of field environments, including competition with native microbiota, fluctuating temperature and moisture, and multi-year crop rotations [73]. Additionally, we primarily used culture-based methods and *16S rRNA* phylogeny, which may underestimate the diversity of uncultured diazotrophs and provide limited resolution of strain-level genomic differences that shape PGP traits and environmental adaptation [74]. The *16S rRNA* gene can only classify strains to the genus level. Subsequent accurate species identification requires confirmation by combined multi-core gene sequencing or genome sequencing. Given that GN8811 is closely related to *Klebsiella quasivariicola*, a species known to be a human pathogen, further safety evaluation of this strain is required, particularly at the whole-genome level [75,76]. The long-term ecological and biosafety implications of releasing genetically modified *Klebsiella* strains into agricultural soils—particularly with enhanced N-fixation under high N—require rigorous assessment, including horizontal gene transfer risks and effects on native microbial communities and greenhouse gas fluxes [77,78,79]. Finally, our findings are limited to early vegetative stages (up to 45 days under nitrogen-free conditions), and that longer-term effects—including impacts on flowering, grain filling, and yield—remain to be investigated in future field studies.

Future research should therefore prioritize multi-location field trials to validate the performance and stability of GN8811 and its engineered derivatives across contrasting soil types, climates and management systems, ideally coupled with 15N-based quantification of BNF contributions. Comparative genomics and transcriptomics of GN8811, GN2003 and other promising isolates could reveal the genetic determinants underlying their high nitrogenase activity, stress tolerance and PGP traits, informing further strain improvement and the design of synthetic consortia. In addition, stacking regulatory modifications with traits that enhance rhizosphere competitiveness, root colonization and formulation stability—such as biofilm formation, exopolysaccharide production, or compatibility with mycorrhizal fungi—may produce next-generation inoculants with greater robustness [80,81,82]. At a broader scale, integrating engineered diazotrophs with precision N management and climate-smart agronomic practices could enable more predictable reductions in synthetic N use while maintaining or enhancing maize yields [83,84,85]. Ultimately, by combining exploration of local microbial diversity with targeted regulatory engineering, this work contributes to the development of reliable, high-performance biofertilizers that support sustainable intensification of maize-based agroecosystems.

## 5. Conclusions

In summary, this study isolated and characterized multiple nitrogen-fixing bacteria from the maize rhizosphere, which possessed various PGP traits and exhibited broad environmental tolerance. Among these, GN8811 (belonging to *Klebsiella*) was a promising candidate. More importantly, through precise genetic manipulation (deleting *nifL* and enhancing *nifA* expression), we successfully constructed engineered strains with higher nitrogen fixation efficiency and enhanced plant growth promotion, providing excellent candidate strains and a novel technological pathway for reducing reliance on chemical nitrogen fertilizers.

## Data Availability

The original contributions presented in this study are included in the article/Supplementary Material. Further inquiries can be directed to the corresponding author.

## Supplementary Materials

The following supporting information can be downloaded at: https://www.mdpi.com/article/10.3390/microorganisms14051067/s1, Figure S1: Schematic diagram of gene knockout and knock-in procedures; Figure S2: Isolates growth status under nitrogen-free and nitrogen-rich conditions; Figure S3: The growth curve of GN8811 derivatives under nitrogen-free conditions; Figure S4: The genetic modified GN8811 and A1501 inoculation effect on maize; Table S1: Strains, plasmids and primers used in this study. References [86,87,88] are cited in the supplementary materials.
