# Supplementary material for: Isolation and Genetic Enhancement of Nitrogen-Fixing Rhizobacteria for Promoting Growth in Maize
Source: Microorganisms. 2026 May 9;14(5):1067. doi: 10.3390/microorganisms14051067 (PMC13209727; doi:10.3390/microorganisms14051067)
Supplement: Supplementary file 1 [file microorganisms-14-01067-s001.zip › microorganisms-4193123-supplementary figures.pdf]

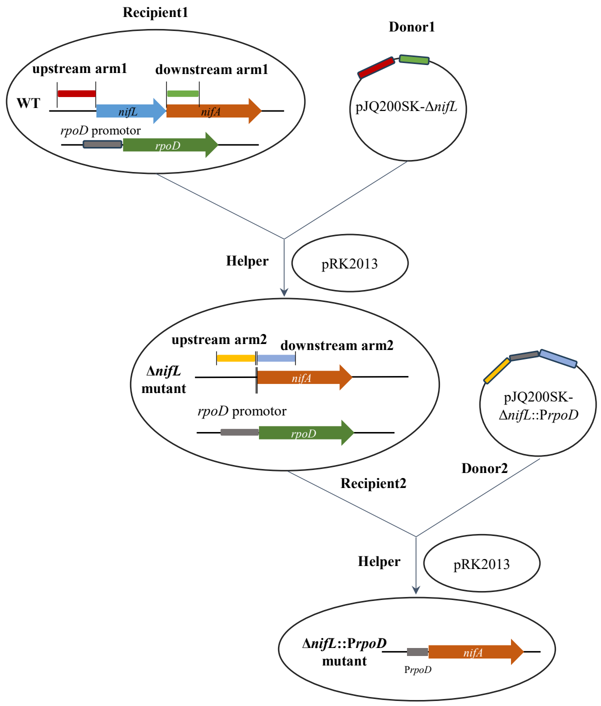


**Figure S1. Schematic diagram of gene knockout and knock-in procedures.**

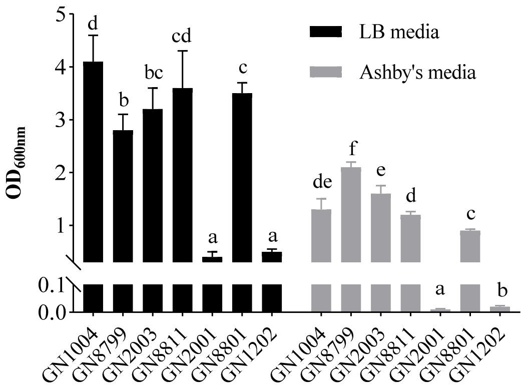


**Figure S2. Isolates growth status under nitrogen-free and nitrogen-rich conditions.** LB medium and Ashby's medium were used to simulate nitrogen-rich and nitrogen-free conditions, respectively. The growth status of all isolates was compared based on OD600 values after 2 d of incubation. The initial inoculum concentration was adjusted to an OD600 of 0.01. Error bars represent SD. Significant differences between means are indicated by different letters based on ANOVA followed by Tukey's test ($\alpha$ = 0.05). At least two additional replications of the experiment were carried out.

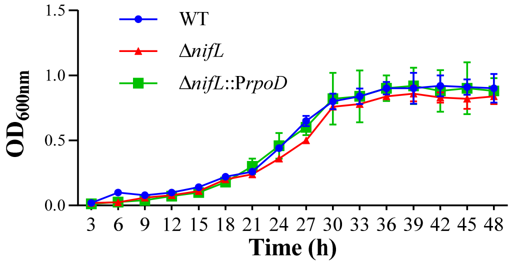


**Figure S3. The growth curve of GN8811 derivatives under nitrogen-free conditions.** Growth curve of GN8811 deritives. WT, wild type; Δ*nifL*, *nifL* mutant; Δ*nifL*::P*rpoD*, *nifL* mutant compensate with *rpoD* promoter. Error bars represent SD. At least two additional replications of the experiment were carried out.

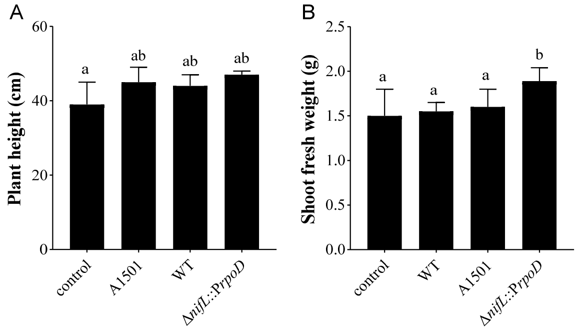


**Figure S4. The genetic modified GN8811 and A1501 inoculation effect on maize.** Plant shoot height (A) and shoot fresh weight (B) were measured to evaluate the effect of the inoculation performance of individual isolates on maize under nitrogen-free condition. Error bars represent SD. Significant differences between means are indicated by different letters based on ANOVA followed by Tukey's test ($\alpha$= 0.05). At least two additional replications of the experiment were carried out.
